# Influence of *β*S-Globin Haplotypes and Hydroxyurea on Arginase I Levels in Sickle Cell Disease

**DOI:** 10.1155/2016/9172726

**Published:** 2016-05-04

**Authors:** J. A. Moreira, R. P. G. Machado, M. R. Laurentino, Romelia Pinheiro Gonçalves Lemes, M. C. Barbosa, T. E. Santos, I. C. J. Bandeira, A. M. C. Martins

**Affiliations:** ^1^Post-Graduate Program in Pathology, Federal University of Ceará, Rua Capitão Francisco Pedro, 1210 Rodolfo Teófilo, 60430-370 Fortaleza, CE, Brazil; ^2^Post-Graduate Program in Development and Technological Innovation in Drugs, Federal University of Ceará, Rua Capitão Francisco Pedro, 1210 Rodolfo Teófilo, 60430-370 Fortaleza, CE, Brazil; ^3^Department of Clinical and Toxicological Analysis, University of Ceará, Rua Capitão Francisco Pedro, 1210 Rodolfo Teófilo, 60430-370 Fortaleza, CE, Brazil; ^4^Post-Graduate Program in Pharmaceutical Sciences, Federal University of Ceará, Rua Capitão Francisco Pedro, 1210 Rodolfo Teófilo, 60430-370 Fortaleza, CE, Brazil

## Abstract

*Introduction.* Sickle cell disease (SCD) is characterized by hemoglobin S homozygosity, leading to hemolysis and vasoocclusion. The hemolysis releases arginase I, an enzyme that decreases the bioavailability of nitric oxide, worsening the symptoms. The different SCD haplotypes are related to clinical symptoms and varied hemoglobin F (HbF) concentration. The aim of this study was to evaluate the impact of the *β*S gene haplotypes and HbF concentration on arginase I levels in SCD patients.* Methods.* Fifty SCD adult patients were enrolled in the study and 20 blood donors composed the control group. Arginase I was measured by ELISA. The *β*S haplotypes were identified by polymerase chain reaction-restriction fragment length polymorphism (PCR-RFLP). Statistical analyses were performed with GraphPad Prism program and the significance level was *p* < 0.05.* Results.* Significant increase was observed in the arginase I levels in SCD patients compared to the control group (*p* < 0.0001). The comparison between the levels of arginase I in three haplotypes groups showed a difference between the Bantu/Bantu × Bantu/Benin groups; Bantu/Bantu × Benin/Benin, independent of HU dosage. An inverse correlation with the arginase I levels and HbF concentration was observed.* Conclusion.* The results support the hypothesis that arginase I is associated with HbF concentration, also measured indirectly by the association with haplotypes.

## 1. Introduction

Sickle cell disease (SCD) is a blood disease characterized by the presence of homozygous hemoglobin S (Hb), which is produced due to a point mutation in the beta-globin gene (a single amino acid substitution) [1].

The deoxygenated HbS polymerization is the primary event in the molecular SCD pathogenesis, resulting in a distortion of the red cell shape and a decrease in its deformability. These rigid cells are responsible for the hemolysis and vasoocclusive phenomena that are the hallmark of the disease [2].

Red blood cell hemolysis releases plasmatic arginase I that catalyzes the hydrolysis of L-arginine, the required substrate for nitric oxide (NO) synthesis, into L-ornithine and urea, thereby contributing to reduction in NO bioavailability and endothelial dysfunction in SCD [3, 4].

Arginase I, which is found predominantly in the liver and kidneys, is also present in human red blood cells and can be induced in many cell types by a variety of cytokines and inflammatory stimulus. In SCD, high plasma arginase I activity is associated with pulmonary hypertension [5].

The SCD presents a heterogeneous clinical course, related to different genetic factors. One of these factors is the presence of specific *β*-globin gene cluster haplotype. These haplotypes are designated as Benin (BEN), Bantu or Central African Republic (BAN or CAR), Senegal (SEN), Cameroon (CAM), and Arab-Indian (ARB) types, according to the geographical area in which they are most commonly found. The different haplotypes are related to varied clinical symptoms and different HbF levels [6, 7].

In this context, the present study aims to evaluate the impact of the *β*S gene haplotypes and the HbF concentration on arginase I levels in adult patients with SCD.

## 2. Material and Methods

### 2.1. Patients

It is a cross-sectional and analytical study with fifty SCD adult outpatients at the University Hospital Walter Cantídio, Fortaleza, Ceará, Brazil. All patients signed an informed consent, according to the protocol approved (number 013.03.12.) by the Ethics Committee of the Federal University of Ceará (UFC). The research has been carried out in accordance with the Code of Ethics of the World Medical Association (Declaration of Helsinki) for experiments involving humans. Eligibility criteria included patients with SCD molecular diagnostics (HbSS), from 18 years of age, in treatment with hydroxyurea (HU) on a dosage of 0.5 to 1.5 g/kg/day for at least six months, with no recent blood transfusion, at baseline and in accordance with the Ballas et al. [8]. Data about HbF concentration and HU dosage were taken from the medical records.

SCD patients were stratified into three groups according to the genotype for the *β*S-globin gene haplotype: Bantu/Bantu (*n* = 26), Bantu/Benin (*n* = 18), and Benin/Benin (*n* = 6).

Control group was composed by twenty blood donors (HbAA), which were healthy individuals without clinical comorbidities, nonsmokers, and nonalcoholic.

### 2.2. Molecular Biological Analysis

DNA was isolated from leukocytes collected in tube with anticoagulant EDTA (ethylenediaminetetraacetic acid), following the protocol of Sambrook et al. [9]. The presence of HbS was confirmed by polymerase chain reaction-restriction fragment length polymorphism (PCR-RFLP), according to the methods of Saiki et al. [10]. The analysis of beta S gene cluster haplotypes was performed by PCR-RFLP, with analysis of six polymorphic restriction sites, according to the methods of Sutton et al. [11]: 5′*γ* ^G^ - Xmn I, *γ* ^G^ - Hind III, *γ* ^A^ - Hind III, *ψβ* - Hinc II, 3′*ψβ* - Hinc II, 5′*β* - Hinf I. The *β*S haplotypes are constructed from the absence (−) or presence (+) of each of the six restriction enzyme sites (Table 1).

### 2.3. Measurement of Arginase I

The serum concentration of arginase I was determined according to the “ELISA kit for human arginase” protocol (USCNK Life Science Inc.). The kit is a sandwich enzyme immunoassay for in vitro quantitative measurement of arginase I in ng/mL. For this test, 6 mL of venous blood was collected in a tube with separator gel, without anticoagulant.

### 2.4. Statistical Analysis

The normality of the data was verified using the Kolmogorov-Smirnov test. Unpaired *t*-test was used for analysis of two numerical variables; Bartlett's test for a multiple comparison of means followed by Tukey's Multiple Comparison posttest. Correlation between arginase and HbF levels was analyzed by Spearman test. The multiple regression model with dummy variables was performed to evaluate the effect of beta-globin haplotypes on arginase levels. All statistical analyses were performed using the GraphPad Prism 5.0 program. Results were expressed as mean ± standard error of the mean (SEM) and *p* < 0.05 was considered statistically significant.

## 3. Results

**Figure 1:**
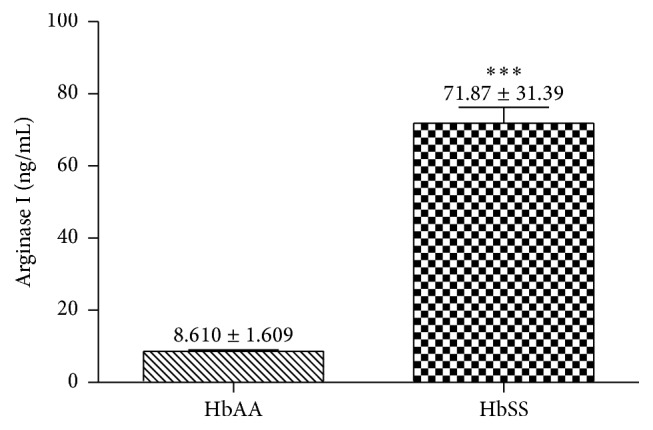
Comparative analysis of arginase I in patients with SCD (HbSS; *n* = 50) compared to the control group (HbAA; *n* = 20). Data values are expressed in mean ± standard error of the mean (SEM) and analyzed by unpaired *t*-test ^*∗∗∗*^ *p* < 0.0001.

We observed a significant increase in the arginase I levels in SCD patients compared with the control group (*p* < 0.0001) (Figure 1).

**Figure 2:**
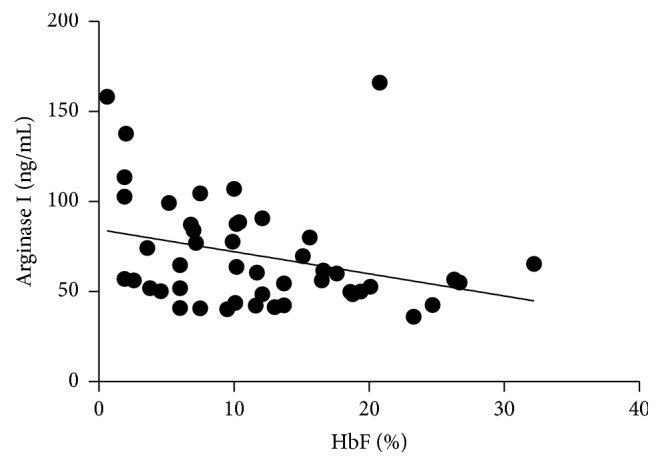
Correlation between HbF concentration and arginase I levels in SCD patients. Results analyzed by Spearman test. *p* = 0.0272; *r* = −0.3222.

The HbF concentration showed an inverse correlation with the arginase I levels (*p* = 0.0272) (Figure 2).

**Figure 3:**
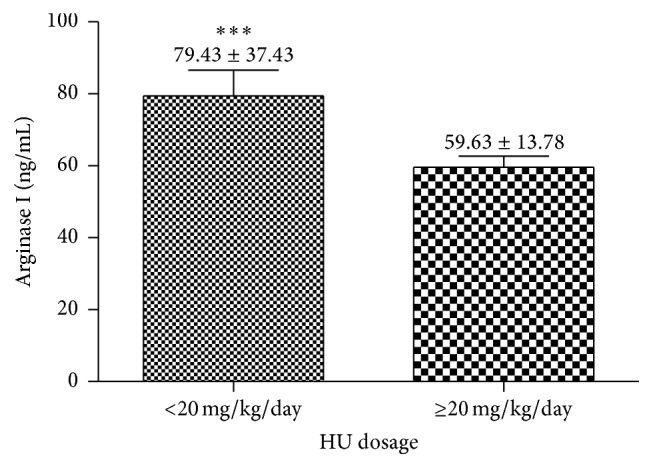
Comparative analysis of arginase I in patients with SCD according to the HU dosage. Data values are expressed in mean ± standard error of the mean (SEM) and analyzed by unpaired *t*-test ^*∗∗∗*^ *p* = 0.0294.

Patients in use of HU in a dose greater than 20 mg/kg/day showed statistical decrease in arginase levels compared to patients at dose usage lower or equal to 20 mg/kg/day (Figure 3). Regarding the HU usage time, we do not get significant differences.

**Figure 4:**
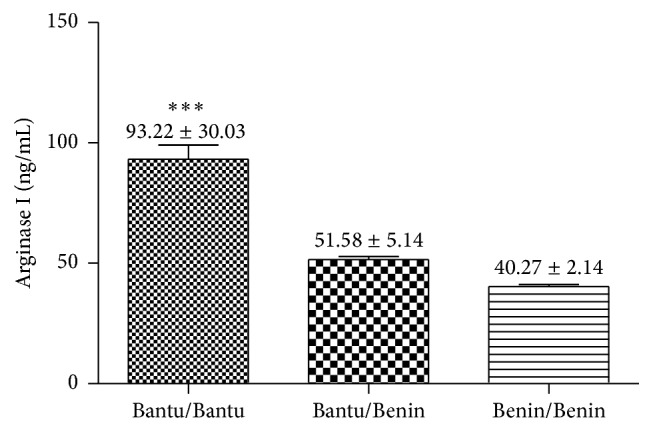
Comparative analysis of arginase I levels in patients with SCD according to the beta-globin haplotypes. Data values are expressed in mean ± standard error of the mean (SEM) and analyzed by Bartlett's test followed by Tukey's Multiple Comparison posttest ^*∗∗∗*^ *p* < 0.001.

**Figure 5:**
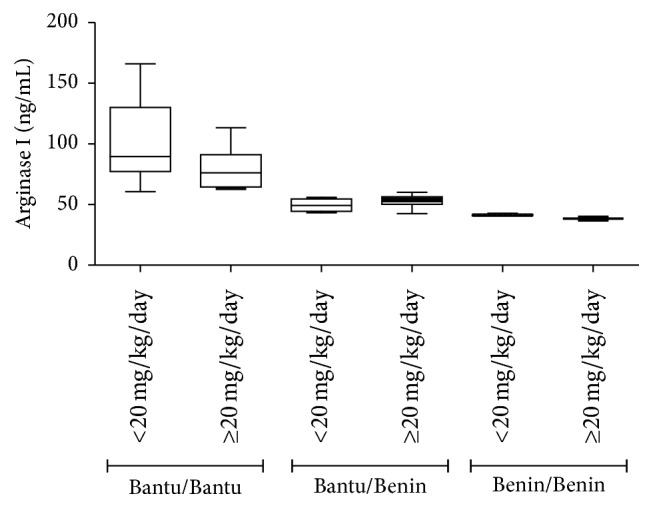
Comparative analysis of arginase I levels in patients with SCD according to the beta-globin haplotypes and HU dosage. Data values are expressed in mean ± standard error of the mean (SEM) and analyzed by unpaired *t*-test. *p* > 0.05 for all comparisons between doses of each haplotype.

Figure 4 shows that patients with haplotype Bantu/Bantu have significantly elevated arginase I levels compared to patients with haplotype Benin/Benin (*p* < 0.001). When we stratified the haplotype groups regarding HU dose, it was observed that the dose of HU does not seem to influence this analysis (*p* > 0.05) (Figure 5).

The regression model with dummy variables analysis showed arginase I levels significantly higher in Bantu/Bantu patients when compared with Benin/Benin patients, independent of the HU dosage (Table 2).

## 4. Discussion

The SCD is characterized by vasoocclusive process, chronic inflammation, and tissue damage. These symptoms are related to low availability of NO that occurs in the disease. The hemolysis of sickle cells releases arginase I, an enzyme that consumes L-arginine, a substrate of NO, limiting the formation of this vasodilator [5, 12, 13]. A significant increase in arginase I plasma levels in SCD patients compared with healthy individuals has been observed. Studies have shown that intravascular hemolysis increases the free arginase I concentration, contributing to the vasoocclusive process.

It was observed that the increase in HbF concentration led to a decrease in the arginase I levels, showing a negative correlation. HbF is the major genetic modulator of the hematologic and clinical features of SCD, inhibiting polymerization of HbS and contributing to the improvement of hemolytic processes and vasoocclusion, which reflects the clinical manifestations of patients [14]. A similar result was found by Iyamu et al. [15]. This study showed a significant decrease in arginase I levels in patients using higher doses of HU. This drug increases the HbF concentration, reducing hemolysis and the consequent release of arginase I [16].

The comparison between the arginase I levels in three groups of haplotypes showed a significant difference between the Bantu/Bantu × Bantu/Benin groups and Bantu/Bantu × Benin/Benin. The results of this study demonstrate that patients with genotype Bantu/Bantu present arginase I concentration statistically higher compared with the other genotypes. The different *β*S haplotypes are related to varied clinical symptoms and HbF levels. In Ceará three types of haplotypes are prevalent: the Benin haplotype is associated with elevated HbF levels and a less serious clinical course of the disease; the Bantu is associated with low HbF levels and more serious symptoms; Bantu/Benin haplotype is associated with moderate HbF levels and intermediate course [17]. Patients with haplotype Bantu/Bantu, due to lower HbF concentration, are less protected against hemolytic processes, reflecting in the increase in plasmatic arginase I levels presented by these patients.

Our findings suggest that SCD patients have increased plasma levels of arginase I, a fact possibly associated with chronic hemolytic process. The HbF concentration differentiates the severity of *β*S haplotypes types and seems to negatively modulate arginase I levels. Further studies, however, are needed to confirm these findings and assess the effect of this biomarker in patients' clinical manifestations.

Whereas *β*S-globin haplotypes play a key role in the clinical manifestations of SCD and response to treatment, their identification should be considered relevant and inserted in clinical practice, as a tool to increase knowledge and management of patients for better monitoring and quality of their lives.

## Figures and Tables

**Table 1:** Genotypes, SNP combinations, and restriction enzyme to determine each haplotype.

Enzyme	Region	DNA fragment size (bp)	DNA fragment after cleavage	Haplotype				
				Bantu	Benin	Senegal	Cameroon	Arab-Indian
Xmn I	5′*γ* ^G^	650	450 + 200	−	−	+	−	+
Hind III	*γ* ^G^	780	430 + 340 + 10	+	−	+	+	+
Hind III	*γ* ^A^	760	400 + 360	−	−	−	+	−
Hinc II	*ψβ*	701	360 + 340 + 1	−	−	+	−	+
Hinc II	3′*ψβ*	590	470 + 120	−	+	+	+	+
Hinf I	3′*β*	380	240 + 140	−	−	+	+	−

**Table 2:** Regression model with dummy variables for the beta-globin haplotypes × arginase, according to HU dosage.

	HU dosage	
	<20 mg/kg/day	≥20 mg/kg/day
Arginase I levels versus beta-globin haplotypes	*r* ^2^ = 0.4842	*r* ^2^ = 0.6177
	*p* = 0.0001	*p* = 0.0001

## Additional Points

This study could not consider a higher sampling due to the low prevalence of the disease in our region. However, about 62.5% of patients treated at the University Hospital Walter Cantídio, Fortaleza, Ceará, Brazil, participated in the study. Furthermore, some patients could not to be enrolled in the study because they were not at baseline state according to Ballas et al. [8]. Another important limitation is that this study did not conduct a follow-up to ensure adherence to treatment with HU for all patients.
